# Expression of CIDE proteins in clear cell renal cell carcinoma and their prognostic significance

**DOI:** 10.1007/s11010-013-1605-y

**Published:** 2013-03-11

**Authors:** Ming Yu, Hui Wang, Jun Zhao, Yuan Yuan, Chao Wang, Jing Li, Lijun Zhang, Liying Zhang, Qing Li, Jing Ye

**Affiliations:** 1 The Department of Pathology, Xijing Hospital, Fourth Military Medical University, Xi’an, Shaanxi China; 2 The Department of Ultrasonography, Xijing Hospital, Fourth Military Medical University, Xi’an, Shaanxi China

**Keywords:** Cideb, Lipid droplet, Clear cell renal cell carcinoma, Metabolism

## Abstract

Clear cell renal cell carcinoma (ccRCC) is the major and aggressive subtype of renal cell carcinoma. It is known to derive its histologic appearance from accumulation of abundant lipids and glycogens. The cell death-inducing DFF45-like effector (CIDE) family has been characterized as the lipid droplet proteins involved in the metabolism of lipid storage droplets. The purpose of this study was to evaluate the expression of CIDE proteins in ccRCC cells and to investigate their prognostic significance. We examined consecutive patients with sporadic ccRCC, who underwent nephrectomy, to measure their mRNA and protein expression of CIDE proteins. We found that Cidec and ADRP expression were significantly up-regulated in ccRCC, compared with normal kidney tissues. Cideb was down-regulated. We also found that Cideb was expressed more in low-grade ccRCC than in high-grade tumors. To further clarify the relationship between Cideb expression and patient prognosis, we evaluated 57 ccRCC patients followed up for 120 months. Reduced ccRCC Cideb expression was associated with a higher Fuhrman nuclear grade. Patients with high Cideb expression had better overall survival rate than those with low expression (*p* < 0.05). Cideb expression was an independent predictor of survival (*p* = 0.001). Although the biologic function of Cideb in ccRCC remains unknown, the expression level of Cideb might be a novel predictor of prognosis in ccRCC.

**Electronic supplementary material:**

The online version of this article (doi:10.1007/s11010-013-1605-y) contains supplementary material, which is available to authorized users.

## Introduction

Renal cell carcinoma (RCC) is the most common malignant tumor in the adult kidney, accounting for about 3 % of human malignancies [1]. On the basis of current genetic knowledge and histologic findings, RCC can be classified into at least four major subtypes: clear cell (ccRCC), papillary, chromophobe, and collecting duct carcinoma [2, 3]. Among them, ccRCC represents the most common subtype (83 %) [4]. Unfortunately, ccRCCs show an extremely variable clinical course, which cannot be predicted. Additional prognostic markers are needed for a more accurate determination of the prognosis and the improvement of therapeutic strategies.

Clear cell RCC is characteristically of a bright golden color, and the clear appearance of tumor cells is due to cellular storage of lipid and glycogen [5–7]. Some epidemiologic studies have shown that obesity is a risk factor for RCC [8, 9]. In general, previous studies suggested that obese patients (with a BMI >30 kg/m^2^) were associated with a high proportion of ccRCC [10–12]. It is apparent that ccRCC cells possess some abnormalities in the metabolism of lipids and glycogen.

The cell death-inducing DFF45-like effector (CIDE) family has been characterized as the crucial lipid droplet (LD) proteins involved in the formation and stabilization of lipid storage droplets [13, 14]. The CIDE family is composed of three members: Cidea, Cideb, and Cidec (CIDE-3 or Fat-specific protein 27) [14]. Previous studies have suggested that Cidea is predominantly expressed in brown adipose tissue [15], and mammary glands [16], while Cidec is expressed at high levels in white adipose tissue [17]. Cideb is strongly expressed in the liver and kidney, in both mice and humans [18, 19].

The presence of abundant LDs suggests that LD proteins are associated with the development of ccRCC. It has been reported adipose differentiation-related protein (ADRP), a LD protein, is highly up-regulated both at the transcriptional and protein levels in ccRCC [20]. The role of CIDE family proteins has not been evaluated. In this study, we measured the CIDE family protein expression in ccRCC using real-time quantitative PCR and western blot. CIDE family expression levels were correlated with the malignancy of ccRCC. We examined 57 consecutive patients with ccRCC to evaluate Cideb expression level in primary tumors and its prognostic significance.

## Materials and methods

### Tissue and antibody

Clear cell RCC and corresponding normal kidney samples were collected from patients who underwent nephrectomy at the Fourth Military Medical University and its affiliated hospitals. Patients did not receive any preoperative therapy. All specimens were snap-frozen with liquid nitrogen and stored at −80 °C for nucleic acid and protein extraction. The histologic slides, stained with hematoxylin and eosin, were reviewed to confirm nuclear Fuhrman grading. Written informed consent was obtained for studying gene expression. The study protocol was approved by the institutional ethics committee. The case series consisted of 10 fresh ccRCC tumors and 10 fresh normal renal tissue specimens obtained from 10 patients. These tumors were classified according to the Fuhrman’s nuclear system. Five patients had low grade tumors (grade 1 and grade 2) and five high grade tumors (grade 3 and grade 4). 57 pathologically confirmed sporadic ccRCC patients, diagnosed from May 2001 to December 2003, were also identified: 15 tumors were grade 1, 18 grade 2, 16 grade 3, and 8 grade 4. The mean follow-up period was 52 months (range: 2–116). The mean tumor size was 6.47 ± 2.97 cm (Mean ± standard deviation). The mean patient age was 59 years (range 32–79).The clinicopathological data are summarized in Table 1. Rabbit anti-Cidea and anti-Cidec polyclonal antibodies were donated by Dr. Peng Li (Department of Biological Sciences and Biotechnology, Tsinghua University, Beijing, China). Mouse anti-Cideb monoclonal was generated by Dr Boquan Jin (Department of Immunology, the Fourth Military Medical University, Shaanxi, China). Anti-GAPDH and anti-β-tubulin antibody were purchased from Abcam (Cambridge, MA, USA).

**Table 1:** Clinical and histopathological characteristics

Fuhrman grade	Patient (%)	Gender		Age (years)	
		Male	Female	Median	Range
Grade 1	15 (14 %)	5	3	53	46–62
Grade 2	18 (48 %)	21	8	58	47–73
Grade 3	16 (23 %)	11	3	56	42–71
Grade 4	8 (15 %)	6	3	58	51–75

### Oil Red O staining

The frozen sections of fresh renal tissues and ccRCC samples were stained with Oil Red O. In brief, cryopreserved tissues were cut into 10-μm sections, and fixed in 10 % formalin for 5 min. Sections were washed in 60 % isopropanol for 2 min, then incubated in Oil Red O (Sigma, USA) working solution for 15 min. The stained tissues were washed using 60 % isopropanol, and then water, to remove residual staining. Slides were counterstained in hematoxylin for 2 min. The slides were mounted with aqueous mounting media and glycerin jelly, and examined under light microscopy.

### Electron microscopy

The tissues for electron microscopy were fixed in 2.5 % cold glutaraldehyde overnight, at 4 °C. Tissues were then rinsed for 1 h in cold phosphate buffer solution (PBS, 0.1 M, pH 7.4) and fixed in 1 % osmium tetroxide for 1 h. Tissues underwent gradient acetone dehydration, were Epon 812 resin embedded, and ultra-thin sections (70 nm) were cut onto slides. Sections were stained with uranyl acetate and lead citrate for JEM-1011 transmission electron microscope observation.

### RNA isolation, cDNA synthesis, and quantitative PCR analysis

RNA was isolated from 10 frozen tumors and 10 normal renal specimens, using Trizol reagent (invitrogen, USA), according to the manufacturer’s instructions. RNA was quantified using the Nanodrop ND-2000 spectrophotometer (Nano-Drop Technologies, Rockland, DE, USA). Total RNA was reverse transcribed using Super-Script II (TaKaRa, Japan). Primers were designed from the sequence of the human cDNAs, and primer sequences are listed in Table 2. Quantitative PCR was performed using the Steponereal-time PCR system (Applied Biosystems) in a total volume of 25 μl with SYBR green (TaKaRa, Japan). cDNAs were serially diluted to obtain five standard solutions that were used in the PCR reaction to generate the reference data. Stepone software was used to generate the reference curve. In each experiment, at least three independent reactions were performed to obtain the mean. Samples were normalized by dividing by the number of copies of GAPDH mRNA.

**Table 2:** Primer sequences

Gene	Primer sequences	Genbank
Cidea	Forward: CATGTATGAGATGTACTCCGTGTC	NM_001279.3
	Reverse: GAGTAGGACAGGAACCGCAG	
Cideb	Forward: AGCCAAAGCATTGGAGACCCTACT	NM_014430.2
	Reverse: TCTGACCAGACTGCAACACCATCA	
Cidec	Forward: TTGATGTGGCCCGTGTAACGTTTG	NM_022094.2
	Reverse: AAGCTTCCTTCATGATGCGCTTGG	
ADRP/perilipin2	Forward: CTGAGCACATCGAGTCACATACTCT	NM_001122.2
	Reverse: GGAGCGTCTGGCATGTAGTGT	
GAPDH	Forward: GAAGGTGAAGGTCGGAGTC	NM_002046.3
	Reverse: GAAGATGGTGATGGGATTTC	

### Immunohistochemistry

We had 57 pathologically confirmed ccRCC patients with long-term follow-up, and obtained the patient’s formalin-fixed paraffin-embedded specimens. All samples were dewaxed in xylene three times for 5–10 min, rehydrated in descending alcohol gradients for 5 min, and blocked for endogenous peroxidase (3 %H_2_O_2_ in 80 %methanol) for 20 min. Antigen retrieval was performed using two treatments in 10 mM sodium citrate in a microwave for 15 min. After blocking non-specific antigen with normal goat serum for 30 min, the slides were incubated with mouse anti-Cideb monoclonal antibody (dilution at 1:200) overnight at 4 °C. Slides were incubated with 100–200 μl of labeled secondary antibody for 30 min at room temperature. Visualization was performed using diaminobenzidine (DAB). The slides were counterstained in hematoxylin for 2 min, dehydrated in ethanol, and mounted. The Cideb expression was evaluated in ccRCC. Low expression was considered as absence or <20 % expression in RCC, and high expression was considered as ≥20 % expression in RCC.

### Western blot

From 10 frozen tumor tissues and 10 normal renal tissues, total samples were separated in a 10 % SDS-PAGE gel and transferred onto a Immobilon-Polyvinylidene fluoride (PVDF) membrane (Millipore Corporation, MA, USA). After blocking with 5 % skimmed milk, the membrane was incubated with mouse anti-Cideb monoclonal antibody (dilution at 1:2,000), rabbit anti-Cidea antibody (dilution at 1:1,000), rabbit anti-Cidec (dilution at 1:4,000), or mouse anti-GAPDH (dilution at 1:1,000). After washing, membranes were incubated for 1 h with a 1:5,000 dilution of peroxidase-conjugated goat anti-rabbit or anti-mouse immunoglobulin (Santa Cruz Biotechnology, Santa Cruz, CA) and expression characterized by chemiluminescence.

### Statistical analyses

Statistical analysis was performed using the SPSS 13.0 software. Statistical analyses were performed with independent samples for *t* test and cox proportional hazards regression model. *p* value <0.05 was considered as statistically significant.

## Results

### Frequent lipid droplet accumulation in ccRCC

**Fig. 1:**
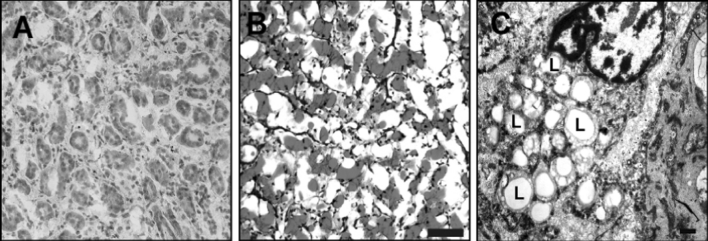
Increased lipid droplets in ccRCC. Images of renal sections stained with Oil Red O from normal renal and ccRCC (**a**, **b**). The *red* *color* spots in Oil Red O staining represent the lipid droplets in the ccRCC(B). *scale* *bars* 50 μm. **c** transmission electron micrograph of renal sections from ccRCC(original magnification ×5000). L,lipid droplets. (Color figure online)

In the light microscope, numerous large Oil Red O-positive, big LDs were visible in ccRCC cells (Fig. 1b), while normal renal tissues contained significantly fewer LDs (Fig. 1a). Similarly, electron microscopy analysis further confirmed the presence of abundant LDs in the samples of clear cell RCC (Fig. 1c).

### mRNA and protein levels of CIDE family in ccRCC

**Fig. 2:**
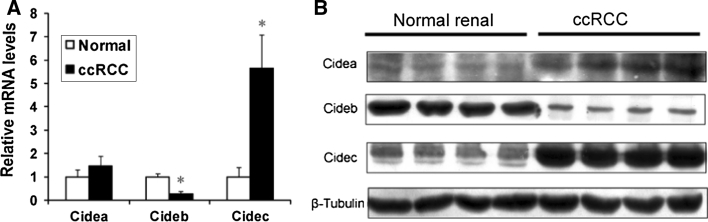
**a** RT-PCR results of CIDE family in ccRCC and adjacent noncancerous tissues. **b** Expression of CIDE protein in ccRCC and adjacent noncancerous tissues. β-tubulin was used as an internal control

We determined CIDE protein and mRNA expression and correlated it with ccRCC clinicopathological parameters. As shown in Fig. 2a, the mRNA level of Cidec was increased nearly sixfold in renal tumor tissues compared with normal renal tissues (*p* < 0.001). In contrast, Cideb mRNA expression in ccRCC decreased about threefold (*p* < 0.001) in comparison with normal renal tissue. mRNA expression of Cidea in ccRCC increased about 1.46-fold (*p* = 0.643). Moreover, ADRP, a PAT member, had about 20-fold higher expression (*p* < 0.001) in ccRCC, compared with the normal renal tissue (data not shown). Similarly, the western blot showed that Cideb protein expression was significantly lower in ccRCC, compared with adjacent normal renal tissues. Cidec protein expression was increased in ccRCC, compared with adjacent normal renal tissues (Fig. 2b). There was no obvious difference in the expressions of Cidea. These results demonstrate that CIDE proteins, especially Cideb and Cidec, correlated with LD accumulation in ccRCC.

### Correlation of Cideb expression with ccRCC grading

**Fig. 3:**
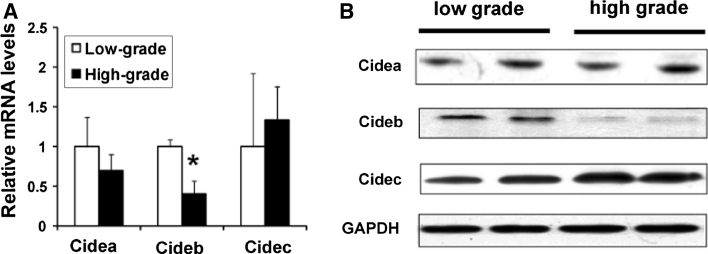
**a** Relative mRNA expression by quantitative real-time PCR analysis.*p = 0.018. The data are shown as mean ± SE. **b** Western blot analysis of low grade and high grade ccRCC

It has been previously demonstrated that ccRCC with lower nuclear grade shows a typical “clear-cell” appearance. As nuclear grade increases, the cytoplasm becomes more eosinophilic and its “clear-cell” character diminishes [21]. Based on nuclear Fuhrman grading, we divided the ccRCC into two groups: low grade (Fuhrman grade 1 and 2) and high grade (Fuhrman grade 3 and 4). When comparing real time-PCR expressions of the CIDE family between low-grade and high-grade tumors (Fig. 3a), a significant difference was found only in Cideb mRNA expression (*p* = 0.018); Cideb mRNA expression was higher in low-grade tumors than in high-grade tumors. There were no significant differences in Cidea and Cidec expressions (*p* = 0.217 and 0.386, respectively). Similar results were found by western blot (Fig. 3b). These data suggest that Cideb is correlated with ccRCC grade.

### Correlation of Cideb expression with the prognosis in ccRCC patients

**Fig. 4:**
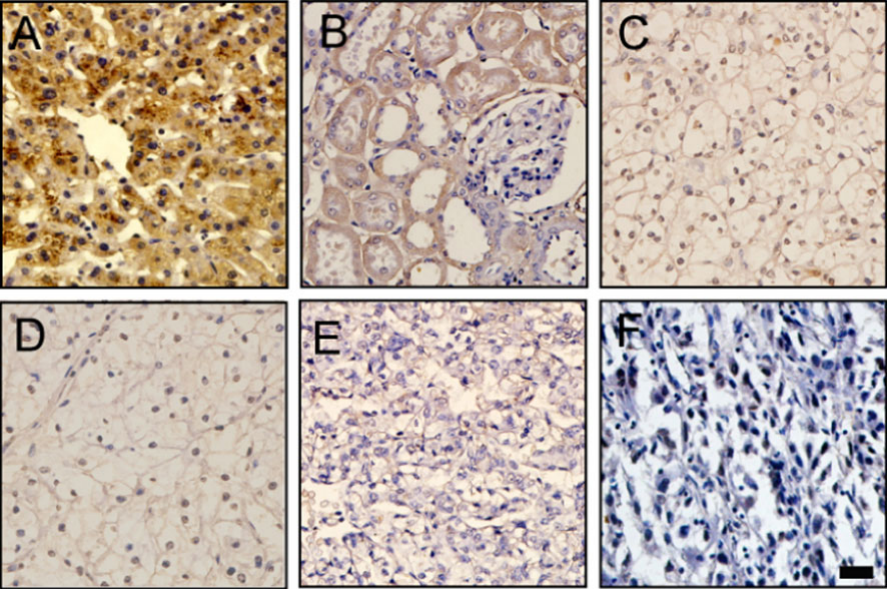
Correlation between Cideb immunostain and Fuhrman nuclear grade in clear cell RCC. **a** liver(positive control) **b** normal renal **c** grade 1 ccRCC **d** grade 2 ccRCC **e** grade 3 ccRCC **f** grade 4 ccRCC (original magnification ×400)

To further demonstrate the prognostic significance of Cideb in ccRCC, immunohistochemistry (IHC) was used to detect the expression level of Cideb in 57 patients with ccRCC. Cideb expression was diffuse and strong staining in normal renal tubular epithelial cells (Fig. 4a). In ccRCC, the cytosolic expression level of Cideb was obviously lower compared with normal renal tissues (Fig. 4b–d). Cideb immunostaining score decreased with the increasing Fuhrman nuclear grade. Fuhrman nuclear grade 1 and grade 2 ccRCC demonstrated high Cideb expression (96.7 %, 30/31), while Fuhrman nuclear grade 3 and grade 4 ccRCC had low expression (92.3 %, 24/26).

**Fig. 5:**
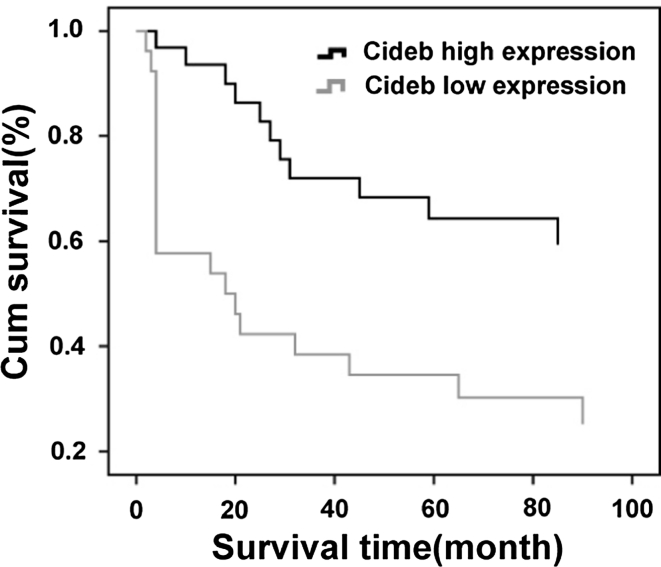
The association of survival with different levels of Cideb expression is illustrated in 57 ccRCC patients (*p* < 0.01). Patients with high expression of Cideb had longer survival than those with low expression of Cideb

To assess the prognostic significance of Cideb expression, we divided the ccRCC patients into two groups: those with high Cideb expression (>20 %), and those with low Cideb expression (absence or <20 % positive cells). Univariate analysis revealed a significantly shorter progression-free survival for patients with low Cideb expression (RR, 2.906; 95 % CI, 1.377–6.132; *p* = 0.005). To evaluate whether low Cideb expression in ccRCC was an independent predictor of overall survival, a multivariate analysis was performed using the Cox proportional hazard test. Low Cideb expression was an independent prognostic factor for survival (*p* = 0.001, Table 3). A Kaplan–Meier curve showed that patients with low Cideb expression in their ccRCC had significantly shorter cancer-specific survival than those with high expression. This difference was apparent very early during follow up (Fig. 5, *p* = 0.005).

**Table 3:** Univariate and multivariate analyses of cancer-specific survival in 57 patients with ccRCC

Characteristics	Univariate analysis			Multivariate analysis		
	RR	95 % CI	*P*	RR	95 % CI	*P*
Age (>60/≤60)	2.365	1.131–4.977	0.022	2.881	1.361–6.097	0.006
Gender (male/female)	0.403	0.140–1.157	0.091	X		
Cideb expression (low/high)	2.906	1.377–6.132	0.005	3.444	1.612–7.356	0.001
Tumor size, cm (≥6/<6)	1.361	0.664–2.791	0.400	X		

*RR* risk ratio, 95 % *CI* 95 % confidence interval

## Discussion

It is well known that the clear appearance of tumor cells results from cellular storage of lipid and glycogen [7]. Clear-cell RCC with lower nuclear grade show a typical “clear-cell” appearance. However, as nuclear grade increases, the “clear-cell” character diminishes, and the number of LD decreases. Some LD proteins (such as ADRP, adipose differentiation-related protein or adipophilin) have been shown to have a role in clear-cell renal carcinoma differentiation [20]. The microvessel density in ccRCC tends to decrease as the tumor grade increases [22, 23].

The CIDE family regulates lipid metabolism and plays an important role in the development of metabolic disorders including obesity, insulin resistance, and hepatic steatosis [15, 17, 24–26]. We identified CIDE family members which were associated with LD storage in ccRCC. Compared with normal kidney tissues, there was significant up-regulation of Cidec and down-regulation of Cideb in ccRCC, but little change in Cidea. Cidea was a BAT-specific marker, while Cidec was most highly expressed in WAT [14]; thus, the different ccRCC expression levels suggest that the lipid storage in ccRCC is more related to WAT than BAT. In addition, we observed that the mRNA levels of Cidec in high-grade ccRCC was slightly higher than that in low-grade ccRCC, but the protein levels of Cidec were similar. Several possibilities might be involved in the regulation of Cidec, such as protein degradation, transcriptional regulation, etc.. Normally, Cideb is expressed at a high level in liver and kidney tissues [18]. Although CIDE proteins have been shown to regulate the biosynthesis and storage of LDs in adipocytes and hepatocytes [14, 18, 25], the function of CIDE proteins in ccRCC has not been determined.

It is well known that ccRCC contains abundant lipids in the cytoplasm, including triglycerides, cholesterol esters, and phospholipids. These lipids impart the typical gross “yellow” appearance [1, 21, 27]. We confirmed the abundant lipid accumulation in ccRCC using Oil Red O and electron microscopy. We found that Cidec protein expression significantly increased, while Cidea expression did not change in ccRCC, compared with normal renal tissue. The different Cidea and Cidec expression levels in ccRCC suggest that lipid storage in ccRCC is more related to WAT than BAT. The down-regulation of Cideb in ccRCC suggests it may prevent the formation of ccRCC cells. We speculated that Cideb could promote lipid secretion in renal cells, similar to its functions in liver cells [18]. These data strongly suggest that the decreasing level of Cideb protein is implicated in lipid uptake and storage in clear-cell RCC. Moreover, Cideb expression levels were likely to reflect microscopic morphologic appearances and the degree of malignancy of clear-cell RCC.

Using IHC, we found that the expression of Cideb in both grade 3 and grade 4 were lower than that in grade 1 and grade 2, while the protein level of Cideb in grade 1 were similar to grade 2. The differences of Cideb in the ccRCC were similar to ADRP [28]. The higher Fuhrman nuclear grade was associated with poor prognosis [29]. Multivariate analysis confirmed that reduction in the expression of Cideb was an independent prognostic factor related to shorter progression-free survival (*p* = 0.001). Thus, low expression of Cideb might be applied as a novel prognostic marker. Collectively, these findings suggest that ccRCC has abnormal lipid metabolism. We observed that the expression of Cideb was largely decreased at both mRNA and protein levels in ccRCC. Our data indicate that the loss of Cideb expression is an important event in lipogenesis and progression of ccRCC.

The mechanism of abnormal lipid metabolism in ccRCC has not been fully elucidated. It is noteworthy that lipogenesis is part of the malignant process in RCC [30]. In this study, we have shown that low Cideb expression is correlated with higher nuclear grade of the ccRCC and poor clinical outcome. A further, larger study should be performed to extend and validate the precise mechanism by which Cideb regulates lipid metabolism and tumor progression in ccRCC.

## Electronic supplementary material

Below is the link to the electronic supplementary material.

Supplementary material 1 (DOCX 20 kb)
