# Supplementary material for: Expression of CIDE proteins in clear cell renal cell carcinoma and their prognostic significance
Source: Mol Cell Biochem. 2013 Mar 11;378(1):145–51. doi: 10.1007/s11010-013-1605-y (PMC3634988; doi:10.1007/s11010-013-1605-y)
Supplement: Supplementary file 1 — Supplementary material 1 (DOCX 20 kb) [file 11010_2013_1605_MOESM1_ESM.docx]

Jan25, 2013

Dr. Naranjan Dhalla

Editor

Molecular and Cellular Biochemistry

Dear Dr. Dhalla:

We are pleased to submit our revised manuscript (mcbi-6362) entitled “Expression of CIDE proteins in Clear Cell Renal Cell Carcinoma and their prognostic Significance” by Yu M,et al. to be considered for publication as an article in the Molecular and Cellular Biochemistry

.

We would like to thank you and reviewers for all the insightful suggestions and comments for thismanuscript.In the revised manuscript, we have performed many changes to the text suggested by reviewers. Major changes in our revised manuscript are as follows.

1．As you have noticed and mentioned in the letter, reviewer 1’s concern “Cidec looks

increased at the protein level but not mRNA”. In addition, we obsereved that Cidec mRNA in high grade ccRCC increased, this upregulation was no difference by statistics(*p*>0.05). Several possibilty might be involved, such as protein degradation, transcriptional regulation. However, the factual reason behind this discrepancy is not clear.

2. As reviewer 1’s concern “In the methods, they state that they had 57 patient samples. Did they look at protein for all of them?”. We select 10 fresh ccRCC tumors and 10 fresh adjoin normal renal tissues to analyse the protein and mRNA levels of CIDE family. For the western blot, we choose a series of representive results. We have 57 pathologically confirmed ccRCC patients with long-term follow-up, and all 57 patient’s formalin-fixed paraffin-embedded specimens were collected. The expression of all samples were detected using immunohistochemistry, and every slide was observed and evalued by three pathologists. These data are shown in revised materials and methods

3．According to your and reviewer 2’s suggestion, we discuss about cideb expression grade. We found that the expression of Cideb proteins in grade 1 were similar to Grade 2 by immunohistochemistry, and the expression of Cideb proteins in the Grade 3 were similar to Grade4. So,we divided ccRCC into the higher grade and lower grade, and this classification were common in the studies on clear cell carcinoma.

4. We would also like to draw your attention that review1 seems to have strong bias. Just like he said;” Cideb is involved in promoting lipogenesis and lipid droplet size in the liver”. However, we don't agree with this view. As well known,Cideb is localized to LDs and the ER, and could promote the lipidation of VLDL particles by interacting with ApoB. Our previous results reveal that more lipids were accumulated in the livers and the isolated hepatocytes of Cideb-*null* mice(*Ye J, et al. Cell metabolism, 2009, 9: 177-190*). It is well known that the clear appearance of tumor cells results from cellular storage of lipid. So, It is consistant with the observation that the reduced expression of Cideb and the accumulation of lipids in clear cell carcinoma.

Overall, as you can see, we have throughly addressed all the concerns raised by the reviewers. Our data are the first to show theexpression of CIDE proteins in clear cell renal cell carcinoma and their prognostic significance. Our study will shed new light on the understanding of the lipids of clear cell renal cell carcinoma. I am confident that our study represents a significant discovery in this field and will be of great interest to a broad range of audience for Molecular and Cellular Biochemistry.

Thank you very much for your time and consideration. I am looking forward to your favorable reply to our manuscript.

Sincerely yours,

Jing Ye, Ph.D.

The department of Pathology

Fourth Military Medical University

Xi’an,710032

CHINA
